# multiDE: a dimension reduced model based statistical method for differential expression analysis using RNA-sequencing data with multiple treatment conditions

**DOI:** 10.1186/s12859-016-1111-9

**Published:** 2016-06-22

**Authors:** Guangliang Kang, Li Du, Hong Zhang

**Affiliations:** Institute of Biostatistics, School of Life Sciences, Fudan University, 2005 Songhu Road, Shanghai, 200438 People’s Republic of China; State Key Laboratory of Genetic Engineering, School of Life Sciences, Fudan University, 2005 Songhu Road, Shanghai, 200438 People’s Republic of China

**Keywords:** RNA-seq, Differential expression, Multiple conditions

## Abstract

**Background:**

The growing complexity of biological experiment design based on high-throughput RNA sequencing (RNA-seq) is calling for more accommodative statistical tools. We focus on differential expression (DE) analysis using RNA-seq data in the presence of multiple treatment conditions.

**Results:**

We propose a novel method, multiDE, for facilitating DE analysis using RNA-seq read count data with multiple treatment conditions. The read count is assumed to follow a log-linear model incorporating two factors (i.e., condition and gene), where an interaction term is used to quantify the association between gene and condition. The number of the degrees of freedom is reduced to one through the first order decomposition of the interaction, leading to a dramatically power improvement in testing DE genes when the number of conditions is greater than two. In our simulation situations, multiDE outperformed the benchmark methods (i.e. edgeR and DESeq2) even if the underlying model was severely misspecified, and the power gain was increasing in the number of conditions. In the application to two real datasets, multiDE identified more biologically meaningful DE genes than the benchmark methods. An R package implementing multiDE is available publicly at http://homepage.fudan.edu.cn/zhangh/softwares/multiDE.

**Conclusions:**

When the number of conditions is two, multiDE performs comparably with the benchmark methods. When the number of conditions is greater than two, multiDE outperforms the benchmark methods.

## Background

High-throughput RNA sequencing (RNA-seq) technologies are emerging rapidly in recent years, which are widely used in biological studies [1]. One of the most important biological problems is to identify differentially expressed genes between multiple experimental conditions. As a result, the key of analyzing these data lies in establishing an appropriate statistical model for RNA-seq count data and consequently preforming differential expression (DE) analysis. Many statistical methods have been developed to fulfill this commission [2, 3]. Methods such as DEGSeq assume that the read count follows a Poisson distribution [4]. Nevertheless, this assumption is violated in the presence of variance overdispersion due to technological and biological variations. An additional parameter can be used to account for the overdispersion. DESeq2 and edgeR, for example, both assume a negative binomial distribution [5, 6]. Much more efficient estimates of the dispersion parameters are obtained using an empirical Bayesian method, resulting in a considerable improvement of DE analysis power and a better control of false positive rate if the sample size is very small. These two methods have been implemented in the Bioconductor packages edgeR and DESeq2, respectively.

In many biological studies, biologists are interested in identifying those genes differently expressed between multiple conditions. For example, the transcriptome of several stages of embryo development or several subtypes of cancer simultaneously enable them to get insight into the sophisticated biological mechanism on a much more comprehensive point [7, 8]. Moreover, the read counts of various conditions can be correlated with each other if they are generated from the same subject. This further complicates the statistical analysis. When the number of conditions, *D*, is greater than two, an analysis of variance (ANOVA) model can be used to detect those genes differentially expressed between the conditions. The chi-squared test based the ANOVA model has *D*−1 degrees of freedom.

To improve power of DE analysis, we recently proposed a 1-df test based on a rank-reduced logistic ANOVA model for logarithm of the expected read count, where the read count was assumed to follow a Poisson-lognormal distribution [9]. This method was termed PLN-ANOVA. In this paper, we present a novel framework committing to facilitate the DE analysis of RNA-seq read count data generated by experiments with multiple conditions, which takes the correlation between samples (if any) into account. The negative binomial distribution is used to model the read count, where the effects of gene and condition on the read counts are incorporated through a two-factor log-linear model with both main effects and interaction effects, while the association between gene and condition is quantified through *D* interaction terms for each of *G* genes. We propose to reduce the dimension of independent interaction parameters from (*D*−1)(*G*−1) to *D*+*G*−2. A rigorous statistical estimation/test procedure is developed in this paper, which could be much more efficient compared with the ones without dimension reduction when *D*>2. The major difference between the current paper and [9] are two-fold: in the distribution assumptions on the read count are different, and the former can deal with both matched samples and unmatched samples but the later can only deal with matched samples.

## Methods

In this section, we describe a rank-reduced model for the RNA-seq read counts of biological samples drawn from multiple conditions, then develop an estimation/test procedure for DE analysis.

### Notation and model

Let *X* denote the read count (i.e., the number of reads mapped to a reference) for any interested gene of any sample. Let *δ* denote the library size factor for that sample, then we can normalize the read count by *Y*=[*X*/*δ*], where [ *a*] is the integer closest to *a*. The size factor can be estimated by any normalization method to be described in the next subsection. As in many statistical methods for analyzing RNA-seq read counts, we assume that *Y* follows a negative binomial distribution. That is, there is an underlying gene expression *Z* that follows a gamma distribution, and *Y* follows a Poisson distribution with expectation *Z*. This way, *Y* marginally follows a compound Poisson-gamma distribution (i.e., negative binomial distribution). In the following, we extend the negative binomial distribution to model the read counts of biological samples drawn from multiple conditions, where the samples can be either independent or correlated with each other.

Consider a study involving *D* conditions, and *n*_*d*_ samples are drawn from the *d*th condition (*d*=1,…,*D*). Let *X*_*idg*_ denote the read count of the *g*th gene for the *i*th sample in the *d*th condition. Let *Y*_*idg*_=[*X*_*idg*_/*δ*_*id*_] be a normalized read count, where *δ*_*id*_ is a size factor for the *i*th sample in the *d*th condition. We assume the following conditions hold:

(C1) *Y*_*i*1*g*_,…,*Y*_*iDg*_ are conditionally independent of each other given their underlying gene expressions *Z*_*i*1*g*_,…,*Z*_*iDg*_, and *Z*_*idg*_ follows a gamma distribution with expectation *μ*_*dg*_ and variance $\phi _{g}\mu _{dg}^{2}$. As a result, *Y*_*idg*_ marginally follows the negative binomial distribution with expectation *μ*_*dg*_ and dispersion parameter *ϕ*_*g*_ (i.e., the variance of *Y*_*idg*_ is $\mu _{dg}+\phi _{g}\mu _{dg}^{2}$).

(C2) The correlation coefficient of $Z_{id_{1}g}$ and $Z_{id_{2}g}$, $\rho _{d_{1}d_{2}}$, is free of gene *g*, which is equal to 0 for *d*_1_≠*d*_2_ in the unmatched situation.

(C3) As in [10] and [9], the following rank-reduced logistic ANOVA model holds for the logarithm of expected gene expression:

1 $$  \log \mu_{dg} = \mu + \alpha_{d} + \beta_{g} + \gamma_{dg} = \mu + \alpha_{d} + \beta_{g} + u_{d}v_{g},  $$

where *μ* is the grand mean, *α*_*d*_ is the main effect for condition *d*, *β*_*g*_ is the main effect for gene *g*, and *γ*_*dg*_:=*u*_*d*_*v*_*g*_ is the interaction effect between gene *g* and condition *d*.

(C4) There are a sufficiently large number of DE genes between *G* genes, i.e., *γ*_*dg*_≠0 for a large number of genes. As remarked below, this implies that *u*_*d*_≠0 for at least one *d*.

Equality restrictions are imposed for the sake of parameter identifiability:

2 $$  \sum_{d=1}^{D} {n_{d}\alpha_{d}} = 0,  $$

3 $$  \sum_{g=1}^{G} \beta_{g} = 0,  $$

and $\sum _{d=1}^{D} n_{d}\gamma _{dg} = 0$, *u*_1_=1, or equivalently

4 $$  \sum_{d=1}^{D} n_{d}u_{d} = 0 \text{ and } u_{1}=1,  $$

5 $$  \sum_{g=1}^{G} v_{g} = 0.  $$

The restrictions (2) and (4) take the sample sizes into account, which will yield simple weighted least squares estimates described in the next section. Under the above restrictions, the main condition effect *α*_*d*_ should be equal to zero so that there is no systematic gene expression difference between the *D* conditions. The main genetic effect *β*_*g*_ quantifies the relative expression of gene *g*. Since $\log \mu _{d_{1}g}-\log \mu _{d_{2}g}=\gamma _{d_{1}g}-\gamma _{d_{2}g}$ provided *α*_*d*_=0, *γ*_*dg*_ quantifies the relative association strength between condition *d* and the expression level for gene *g*. If there is at least one gene differentially expressed between the *D* conditions, then *γ*_*dg*_≠0 for at least one *g*, hence *u*_*d*_≠0 for at least one *d* under decomposition (1). Therefore, the relative association strength can be measured by *v*_*g*_, and the null hypothesis that the *g*th gene is differentially expressed between the *D* conditions can be formulated by

6 $$  H_{g}: v_{g} = 0.  $$

The key idea of model (1) is to approximate the interaction effect *γ*_*dg*_ using the product of two terms that depend on *d* and *g* separately. This reduces the dimension of test problem from *D*−1 (the corresponding null hypothesis is *H*_*g*_:*γ*_1*g*_=…=*γ*_*Dg*_) to one (the corresponding null hypothesis is *H*_*g*_:*v*_*g*_=0) for gene *g*. Obviously, the resultant test could be much more powerful than the chi-square test based on the ANOVA model without such decomposition if *D*>2.

### Read count normalization

In this subsection, we consider the calculation of the size factor *δ*_*id*_. It is widely known that raw counts are not directly comparable between genes due to differential gene lengths and sequencing depths, and reads per kilobase per million reads (RPKM) can be used to correct the resultant technical bias [11]. In DE analysis between multiple conditions, the gene length does not affect the analysis result since such DE analysis focuses on the same gene. However, the condition comparison could greatly suffer from sample specific effects such as sequencing depth and sample specific GC-content effect. The sample specific GC-content effect could arise if two or more samples are sequenced in the same lane. Several within-lane normalization methods (i.e., regression normalization, global-scaling normalization, and full-quantile normalization) can be used to correct the resultant technical bias [12]. On the other hand, such effect can be absorbed into sample specific sequencing depth if only a single sample is sequenced in each lane, and the following four between-lane normalization methods are designed for correcting the technical bias due to sequencing depth.

The first one is the median normalization, denoted by MEDIAN, which takes the form [5]

7 $$  \delta_{id} =\frac{m_{id} }{\left(\prod_{d'=1}^{D}\prod_{i'=1}^{n_{d'}} m_{i'd'}\right)^{\left(\sum_{d'=1}^{D} n_{d'}\right)^{-1}}},  $$

where *m*_*id*_ is the sample median of {*X*_*id*1_,…,*X*_*idG*_}.

The second one is the total count normalization, denoted by TOTAL, which takes the form [13]

8 $$  \delta_{id} = \frac{s_{id} }{(\sum_{d'=1}^{D} n_{d'})^{-1} \sum_{d'=1}^{D}\sum_{i'=1}^{n_{d'}} s_{i'd'} },  $$

where *s*_*id*_ is the sum of {*X*_*id*1_,…,*X*_*idG*_}.

The third one is the quantile normalization, denoted by QUANTILE, which takes the form [2]

9 $$  \delta_{id} = \frac{q_{id}}{(\sum_{d'=1}^{D} n_{d'})^{-1} \sum_{d'=1}^{D}\sum_{i'=1}^{n_{d'}} q_{i'd'}},  $$

where *q*_*id*_ is the 75th percentile of {*X*_*id*1_,…,*X*_*idG*_}.

The fourth is a weighted trimmed mean of M-values, denoted by TMM, which has been implemented in the Bioconductor package edgeR [14]. For sample *i* in condition *d* and sample *i*^′^ in condition *d*^′^, the log-fold changes (M-values) are defined as

$$M_{idg}^{i'd'} = \frac{\log_{2} (X_{idg}/N_{id})}{\log_{2} (X_{i'd'g}/N_{i'd'})},\quad g=1,\ldots,G, $$

where $N_{id}=\sum _{g=1}^{G} X_{idg}$. If one uses sample *i*_0_ in condition *d*_0_ as a reference sample, then the size factor for sample *i* in condition *d* is defined as

10 $$  \delta_{id} = \frac{\sum\limits_{g\in{G^{\ast}}} w_{idg}^{i_{0}d_{0}}M_{idg}^{i_{0}d_{0}}}{\sum\limits_{g\in{G^{\ast}}} w_{idg}^{i_{0}d_{0}}},  $$

where *G*^∗^ is the set of those genes with the upper and lower 30 % of the M-values removed. In edgeR that implements TMM, the sample with upper quartile closest to the mean upper quartile across all samples is used as the default reference. To minimize the variance of *δ*_*id*_, here the weight $w_{idg}^{i_{0}d_{0}}$ is defined as inverse of the estimated variance of M-values:

$$w_{idg}^{i_{0}d_{0}}=\frac{N_{id}-X_{idg}}{N_{id}X_{idg}}+\frac{N_{i_{0}d_{0}}-X_{i_{0}d_{0}g}}{N_{i_{0}d_{0}}X_{i_{0}d_{0}g}}. $$

Using any of the above four normalization methods, the read count *X*_*idg*_ can be normalized by

$$Y_{idg} = \left[\frac{X_{idg}}{\delta_{id}}\right]. $$

### Parameter estimation

In this subsection, we derive estimators of *μ*, *α*_*d*_, *β*_*g*_, *u*_*d*_, *v*_*g*_, *ϕ*_*g*_, and $\rho _{d_{1}d_{2}}$. We first describe two existing methods for estimating *ϕ*_*g*_. Then we develop a simple estimation equation method for estimating *μ*, *α*_*d*_, *β*_*g*_, *u*_*d*_, and *v*_*g*_. Finally, we propose a method for estimating $\rho _{d_{1}d_{2}}$ in the matched sample situation.

First, we estimate the dispersion parameter *ϕ*_*g*_ using an empirical Bayes method or a parametric method via a robust gamma-family generalized linear model. The two dispersion estimation methods have been implemented in the Bioconductor packages edgeR and DESeq2, respectively.

Next, we describe a novel method for estimating *μ*, *α*_*d*_, *β*_*g*_, *u*_*d*_, and *v*_*g*_. Denote *η*_*dg*_= log*μ*_*dg*_, which can be estimated by

11 $$  \hat{\eta}_{dg} = \log \hat{\mu}_{dg} = \log \left(\frac{1}{n_{d}} \sum_{i=1}^{n_{d}} Y_{idg}\right).  $$

Under restrictions (2)-(5), we have the moment estimators of *μ*, *α*_*d*_, *β*_*g*_, and *γ*_*dg*_:

12 $$ \hat{\mu} = \frac{\sum_{d=1}^{D}\sum_{g=1}^{G} n_{d}\hat{\eta}_{dg}}{G\sum_{d=1}^{D} n_{d}},  $$

13 $$ \hat{\alpha}_{d} = \frac{1}{G} \sum_{g=1}^{G} \hat{\eta}_{dg} - \hat{\mu},  $$

14 $$ \hat{\beta}_{g} = \frac{\sum_{d=1}^{D} n_{d}\hat{\eta}_{dg}}{\sum_{d=1}^{D} n_{d}} - \hat{\mu},  $$

$$\hat{\gamma}_{dg} = \hat{\eta}_{dg} - \hat{\mu} - \hat{\alpha}_{d} - \hat{\beta}_{g}. $$

Here we adopt a weighted least squares approach to estimating *u*_*d*_ and *v*_*g*_ [15]. Noting that $\hat {\gamma }_{dg}$ has an asymptotic variance proportional to $n_{d}^{-1}$, we can minimize the weighted sum of squares

$$ l(u_{d},v_{g}) = \sum_{d=1}^{D}\sum_{g=1}^{G} n_{d}(\hat{\gamma}_{dg}-u_{d}v_{g})^{2} $$

subject to restrictions (2)-(5). The resultant solution {$\hat u_{d}: d=1,\ldots,D; \hat v_{g}: g=1,\ldots,G$} satisfies the following equations:

15 $$  \hat{u}_{d} =\frac{\tilde{u}_{d}}{\tilde{u}_{1}} \text{with}~\tilde{u}_{d}= \frac{\sum_{g=1}^{G} \hat{\gamma}_{dg}\hat{v}_{g}}{\sum_{g=1}^{G} \hat{v_{g}^{2}}},  $$

16 $$ \hat{v}_{g} = \frac{\sum_{d=1}^{D} \hat{\gamma}_{dg}n_{d}\hat{u}_{d}}{\sum_{d=1}^{D} n_{d}\hat{u_{d}^{2}}}.  $$

In real situations, most genes are equally expressed with *v*_*g*_=0 and the corresponding information is pure noise for estimating *u*_*d*_. To eliminate the impact of such noise, we can modify (15) as

17 $$ \hat{u}_{d} =\frac{\tilde{u}_{d}}{\tilde{u}_{1}} \text{with}~\tilde{u}_{d}= \frac{\sum_{g\in S} \hat{\gamma}_{dg}\hat{v}_{g}}{\sum_{g\in S} \hat{v_{g}^{2}}},  $$

where *S* is a DE gene set determined by any existing method such as edgeR. Equations (16) and (17) can be easily solved in an iterative manner, and the algorithm for all genes can be greatly sped up via vectorization.

Finally, we derive an estimator of the correlation coefficient $\rho _{d_{1}d_{2}}$ in the matched sample situation. By conditions (C1) and (C2), we have that

$$\begin{array}{@{}rcl@{}} \text{corr}(Y_{id_{1}g},Y_{id_{2}g})=\frac{\text{cov}(Z_{id_{1}g},Z_{id_{2}g})}{\{\text{var}(Y_{id_{1}g})\text{var}(Y_{id_{2}g})\}^{1/2}}\\ =\frac{\rho_{d_{1}d_{2}}\mu_{d_{1}g}\mu_{d_{2}g}\phi_{g}}{\{\mu_{d_{1}g}+\mu_{d_{1}g}^{2}\phi_{g})(\mu_{d_{2}g}+\mu_{d_{2}g}^{2}\phi_{g})\}^{1/2}}. \end{array} $$

Since *Y*_1*dg*_,…,*Y*_*ndg*_ are identically distributed, we can estimate $\text {corr}(Y_{id_{1}g},Y_{id_{2}g})$ with the sample correlation coefficient of $(Y_{1d_{1}g},Y_{1d_{2}g}),\ldots,(Y_{nd_{1}g},Y_{nd_{2}g})$ times a continuity correct term proposed by [16], which is denoted by $r_{d_{1}d_{2}g}$. Here the continuity correct term is used to reduce the estimation bias due to a very small sample size. An estimator of $\rho _{d_{1}d_{2}}$ takes the form

$$\begin{array}{@{}rcl@{}} \frac1G\sum_{g=1}^{G}\frac{r_{d_{1}d_{2}g}\{(\hat\mu_{d_{1}g}+\hat\mu_{d_{1}g}^{2}\hat\phi_{g})(\hat\mu_{d_{2}g}+\hat\mu_{d_{2}g}^{2}\hat\phi_{g})\}^{1/2}}{\hat\mu_{d_{1}g}\hat\mu_{d_{2}g}\hat{\phi_{g}}}. \end{array} $$

To construct a Wald test statistic for testing *H*_*g*_:*v*_*g*_=0, we need to estimate the variance of $\hat v_{g}$, as detailed in the next subsection.

### Variance estimation and Wald test

Since $\hat \mu $, $\hat {\alpha }_{d}$, and $\hat u_{d}$ use the information across a sufficiently large number of genes, it is reasonable to assume that their variances are ignorable compared with those of the other estimators. Therefore, we have

$$\text{var}(\hat v_{g})\approx \frac{\text{var}(\sum_{d}\hat{\eta}_{dg}n_{d}\hat u_{d})}{(\sum_{d}n_{d}\hat {u_{d}^{2}})^{2}}. $$

Using the delta method, we have that

$$\text{var}(\hat \eta_{dg}) \approx \mu_{dg}^{-2}\text{var}(\hat{\mu}_{dg}) = n_{d}^{-1}(\hat{\mu}_{dg}^{-1}+\hat{\phi}_{g}). $$

Therefore, we can estimate $\text {var} (\hat v_{g})$ by

18 $$ \widehat{\text{var}}(\hat v_{g}) = \frac{\sum_{d=1}^{D} n_{d}\hat{u}_{d}^{2}(\hat{\mu}_{dg}^{-1}+\hat{\phi}_{g})}{(\sum_{d=1}^{D} n_{d}\hat{u}_{d}^{2})^{2}}  $$

in the unmatched sample situation. In the matched sample situation with *n*_*d*_=*n* for *d*=1,…,*D*, we need to further estimate $\text {cov}(\hat {\eta }_{d_{1}g},\hat {\eta }_{d_{2}g})$ for *d*_1_≠*d*_2_. The resultant estimate of $\text {var}(\hat v_{g})$ in the matched sample situation, $\widehat {\text {var}}({\hat {v}_{g}})$, takes the form

19 $$\begin{array}{@{}rcl@{}}  \bigg\{n\bigg(\sum_{d=1}^{D} \hat{u}_{d}^{2}\bigg)^{2}\bigg\}^{-1} \bigg\{\sum_{d=1}^{D} \hat{u}_{d}^{2}(\hat{\mu}_{dg}^{-1}+\hat{\phi}_{g})\\ +2\sum_{1\leq{d_{1}}<d_{2}\leq{D}} \hat{u}_{d_{1}}\hat{u}_{d_{2}}\hat{\phi}_{g}\hat{\rho}_{d_{1}d_{2}} \bigg\}. \end{array} $$

With the estimator (18) or (19), we can construct a Wald test statistic

20 $$ T_{g} = \frac{\hat{v}_{g}^{2}}{\widehat{\text{var}}(\hat{v}_{g})},  $$

whose null limiting distribution is the chi-squared distribution with one degree of freedom.

## Results

To evaluate the performance of the proposed method, we conducted both simulation studies and real data analyses. For comparison purpose, we considered two benchmark methods edgeR (version 3.12.1) and DESeq2 (version 1.10.1). The functions *estimateGLMTagwiseDisp* and *estimateDispersions* were used to estimate tagwise negative binomial dispersions in edgeR and DESeq2, respectively. In these two methods, the likelihood ratio tests were used for DE analysis and the default parameters were adopted. We also considered our recently developed method PLN-ANOVA, which was designed for matched samples. In both simulation studies and real data applications for multiDE, the dispersion estimates given by edgeR and DESeq2 produced DE analysis results that were very close to each other. Therefore, in the following studies, we only present the results of multiDE with the dispersion estimates given by edgeR.

### Simulation studies

We considered the situation where only a single sample was sequenced in each lane, as commonly done in real applications, so that the effect of sample specific GC-content can be absorbed into the library size. First we evaluated the four normalization methods using simulation data generated from a real dataset. Then we conducted a comparison study between two benchmark methods (i.e., edgeR and DESeq2) and multiDE equipped with MEDIAN using simulation data generated under model (1). Finally, we conducted a sensitivity analysis by generating data without the assumption of model (1).

#### Simulation based on a real dataset

In multiDE, any of the four methods (namely, MEDIAN, TOTAL, QUANTILE, and TMM) can be used to normalize the read counts.

We generated simulation data using a real data set from an embryonic stem cells study described in the next subsection. In this study, RNA-seq read counts at 6,526 genes were available from nine unrelated individuals. Three different conditions were considered, and each condition had three individuals. Refer to the next subsection for detailed description of this study. We randomly permutated the condition information for the nine samples, then randomly selected 600 DE genes. In each of these DE genes, the read counts of the second condition were the original ones multiplied by a factor of 1.1, and the read counts of the third condition were the original ones multiplied by a factor of 0.9. The rest 5,926 genes were regarded as equally expressed genes. Such experiment was repeated for 400 times, and the four normalization methods were applied to these datasets.

**Fig. 1:**
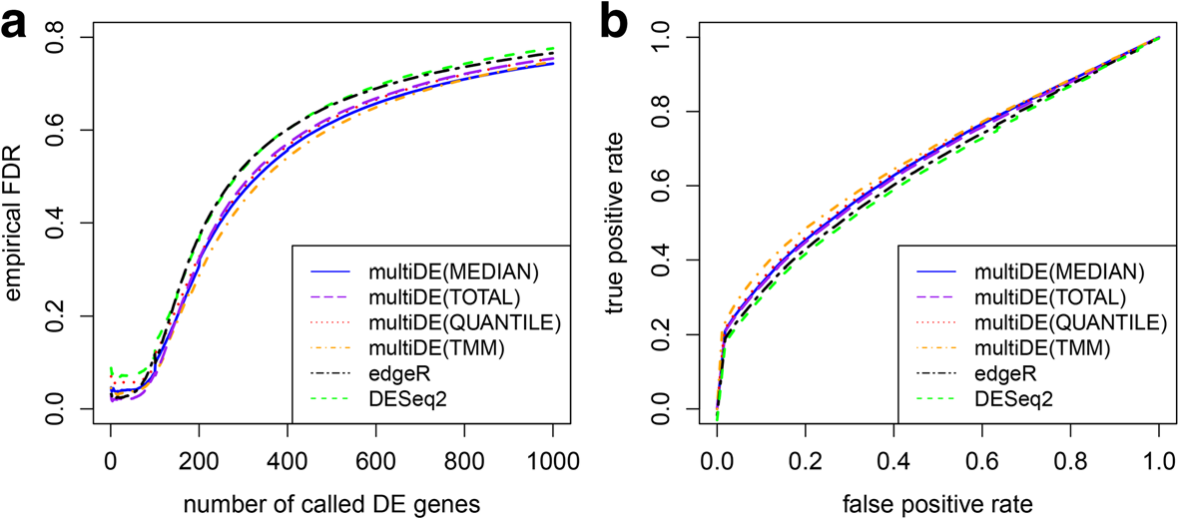
Performance of various methods with simulation data based on the embryonic stem cells study. **a** False discovery rates; **b** ROC curves

The simulation results of multiDE equipped with the four normalization methods were summarized through receiver operating characteristic (ROC) curves and false discovery rates (FDRs) (Fig. 1). In terms of both FDRs and ROC curves, TMM slightly outperformed the other three normalization methods. As stated in [14], TMM is robust against outlying read counts and a significant proportion of DE genes, while the other three normalization methods assume that most genes are not differentially expressed. Actually, these three methods performed relatively poorer than TMM in the application to the above datasets that have about 10 % DE genes. This agrees with the simulation results of [14].

We also applied the two benchmark methods edgeR and DESeq2 to the above simulation datasets. Evidently, multiDE outperformed edgeR and DESeq2 even using the MEDIAN (Fig. 1), we therefore focus on this normalization method in the next two subsections.

#### Simulation based on model (1)

We considered two situations for generating the read count *X*_*idg*_, one was for unmatched samples and the other was for matched samples. The parameters shared the two situations were set to be the same. We let *D* = 2, 3, 4, or 5, and fixed the total number of genes to be *G*=10^4^. Then we set *n*_*d*_=*D*+*d*−1 for *d*=1,…,*D* in the unmatched sample situation, and *n*=4 in the matched sample situation.

To generate RNA-seq read count data, we set *μ*=4, *α*_*d*_=0 for *d*=1,…,*D*,

$$(u_{1},\ldots,u_{D}) =\left\{\begin{array}{lc} (1,-\frac{2}{3}),& D=2 \\ (1,\frac{3}{4},-\frac{6}{5}),& D=3 \\ (1,\frac{4}{5},-\frac{3}{6},-\frac{5}{7}),& D=4 \\ (1,\frac{5}{6},\frac{3}{7},-\frac{5}{8},-\frac{8}{9}),& D=5 \end{array} \right. $$

in the unmatched sample situation, and

$$(u_{1},\ldots,u_{D}) =\left\{\begin{array}{lc} (1,-1),& D=2 \\ (1,0.2,-1.2),& D=3 \\ (1,0.4,-0.6,-0.8),& D=4 \\ (1,0.5,0,-0.5,-1),& D=5 \end{array} \right. $$

in the matched sample situation. We let *v*_*g*_=0 for equally expressed genes *g*=1,…,9000, *v*_*g*_∼−|*N*(0,0.32)| for up-regulated genes *g*=9001,…,9500, and *v*_*g*_∼|*N*(0,0.32)| for down-regulated genes *g*=9501,…,10000. In addition, we randomly generated size factors *δ*_*id*_ from the log-normal distribution with mean and varariance parameters 0 and 0.25^2^, main genetic effects *β*_*g*_ from the normal distribution with mean 0 and variance 0.25^2^, dispersion parameters *ϕ*_*g*_ from the gamma distribution with shape parameter 5 and rate parameter 20, where the shape and rate parameters were close to those for the embryonic stem cells data to be analyzed in the next subsection.

With the above parameters, we then generated underlying gene expressions (*Z*_*i*1*g*_,…,*Z*_*iDg*_) from multivariate gamma distribution with correlation parameters $\{\rho _{d_{1}d_{2}}:1\leq d_{1}\leq d_{2}\leq D\}$ using the Bioconductor package copula [17], and generated the read counts *X*_*idg*_ from the Poisson distribution with expectation *Z*_*idg*_. We let $\rho _{d_{1}d_{2}}=0$ in the unmatched situation and $\rho _{d_{1}d_{2}}\sim U(0.2, 0.4)$ in the matched situation.

**Fig. 2:**
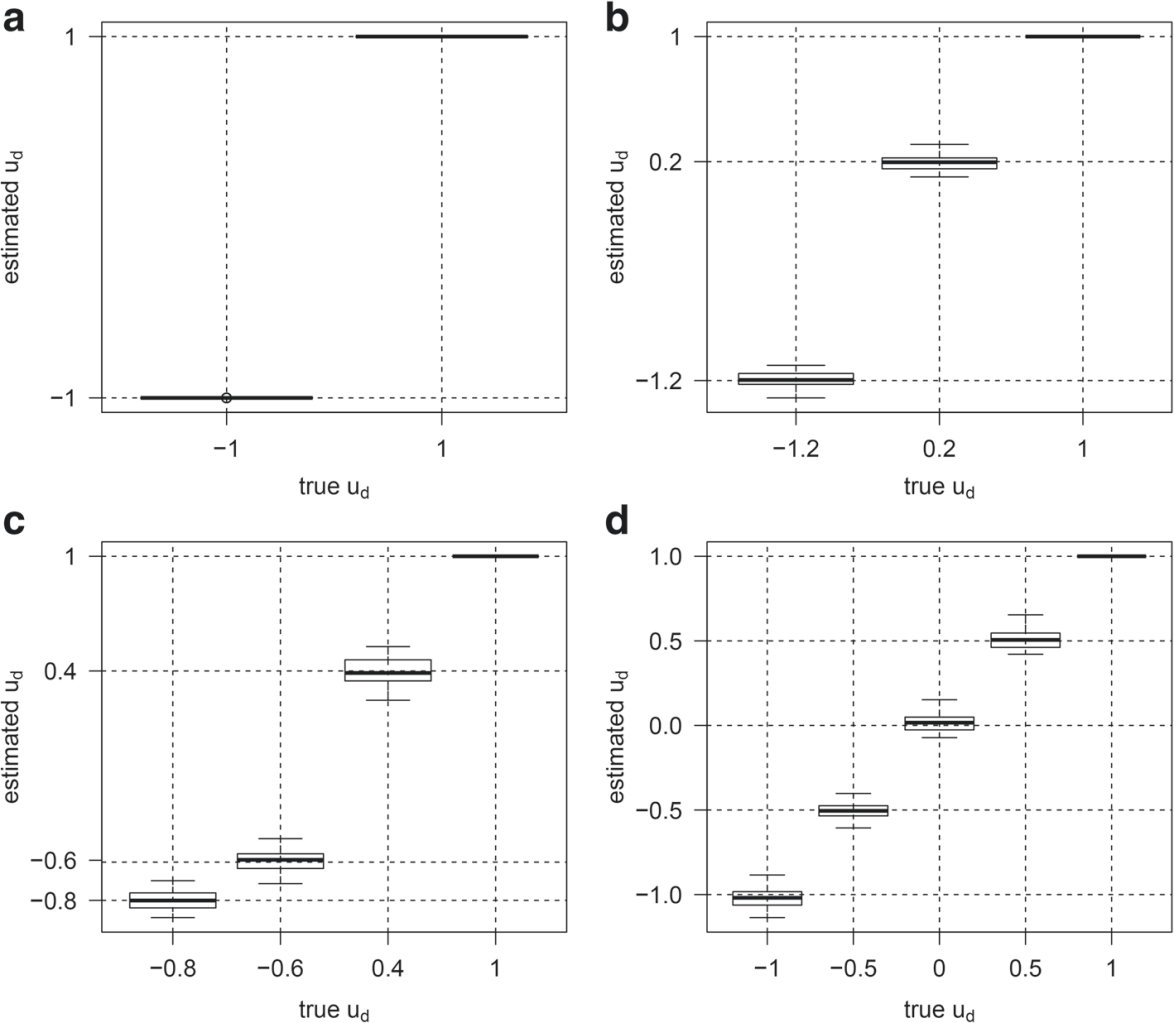
Estimates of *u*_*d*_ in matched sample situation. **a** D=2; **b** D=3; **c** D=4; **d** D=5

**Fig. 3:**
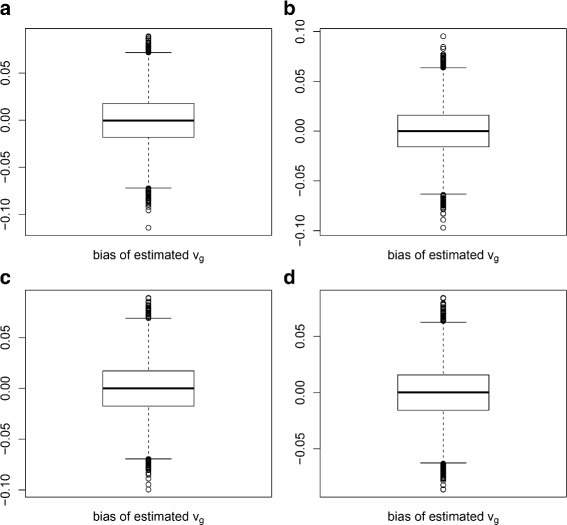
Estimation biases of *v*_*g*_ in matched sample situation. **a** D=2; **b** D=3; **c** D=4; **d** D=5

**Fig. 4:**
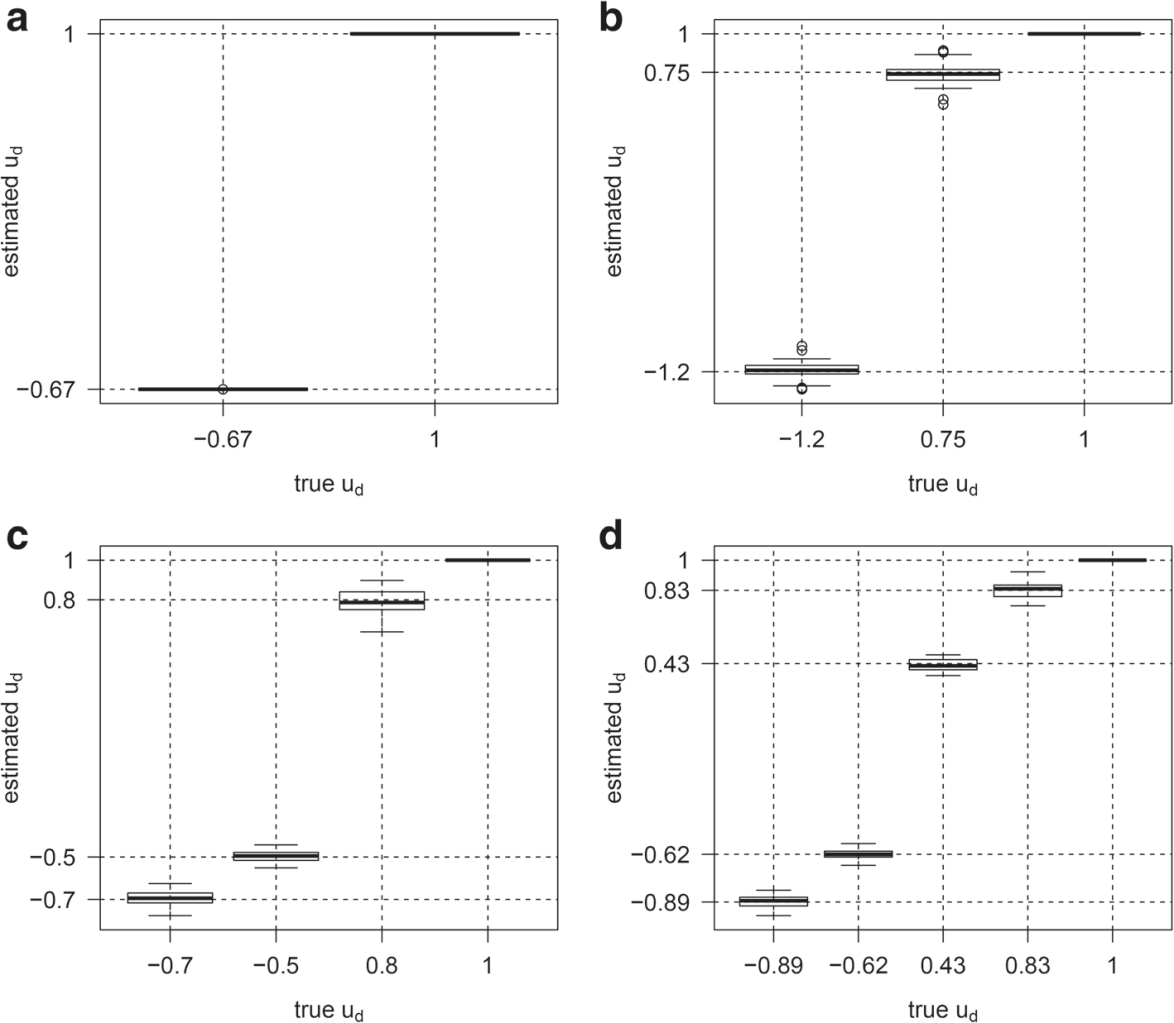
Estimates of *u*_*d*_ in unmatched sample situation. **a** D=2; **b** D=3; **c** D=4; **d** D=5

**Fig. 5:**
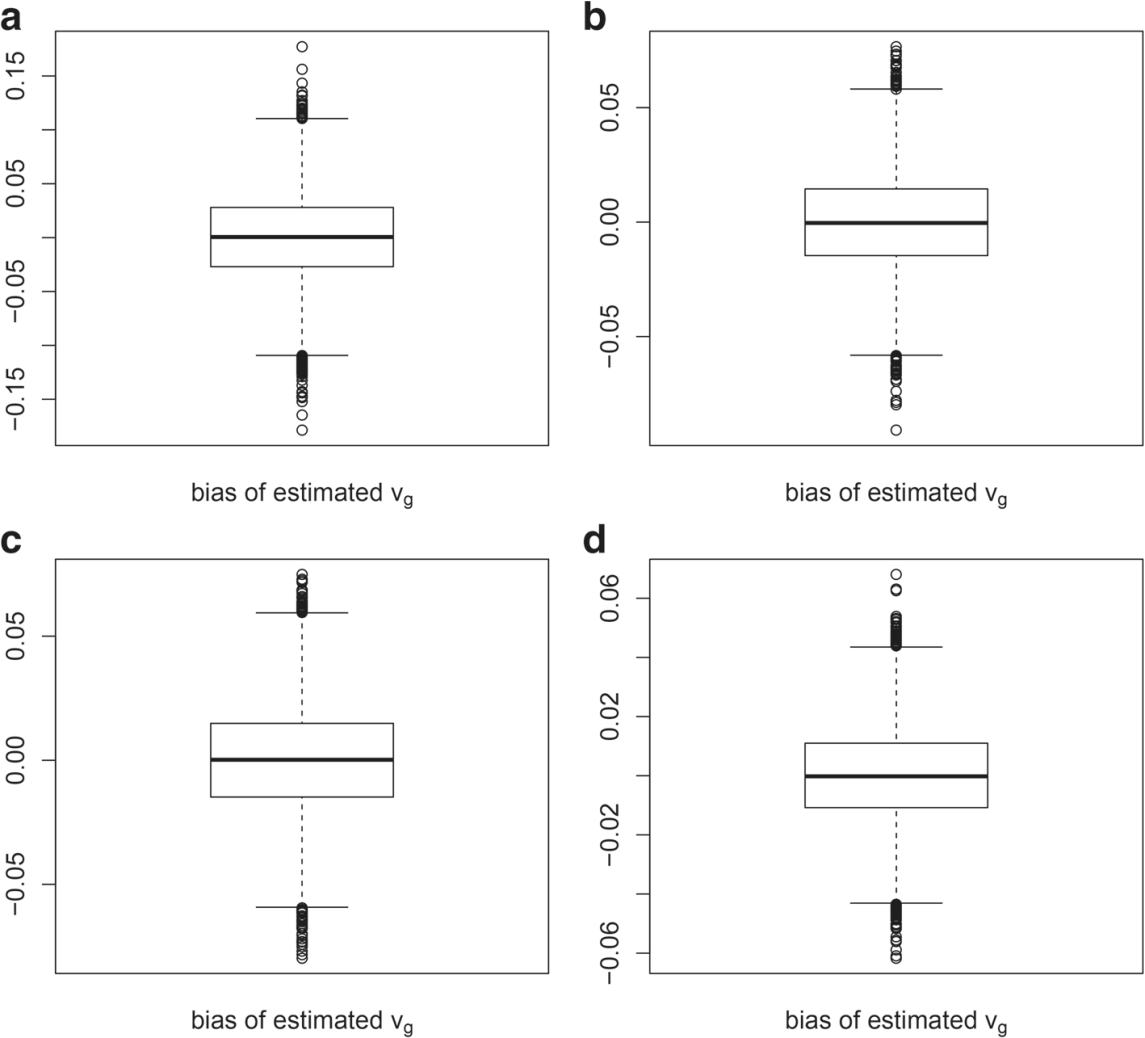
Estimation biases of *v*_*g*_ in unmatched sample situation. **a** D=2; **b** D=3; **c** D=4; **d** D=5

For each parameter combination, we generated 50 datasets. For each dataset, *u*_*d*_ and *v*_*g*_ were estimated using multiDE. To evaluate the estimation accuracy of multiDE, we report in Figs. 2, 3, 4 and 5 the *u*_*d*_ estimates and the biases of *v*_*g*_ estimates in both matched and unmatched sample situations. Overall, the *u*_*d*_ estimates were virtually close to the true ones and the estimation biases of *v*_*g*_ were minor.

**Fig. 6:**
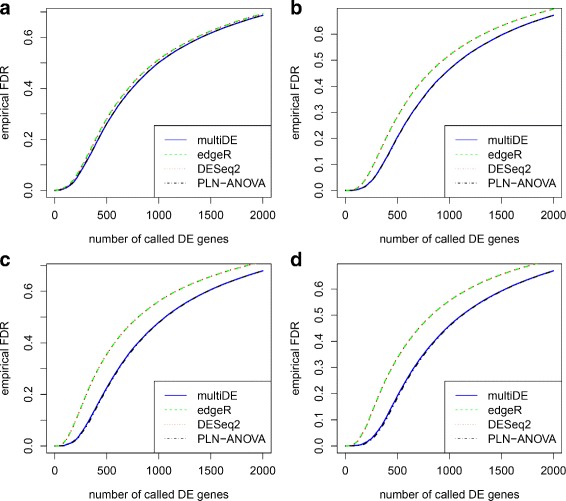
False discovery rates in matched sample situation. **a** *D*=2; **b** *D*=3; **c** *D*=4; **d** *D*=5

**Fig. 7:**
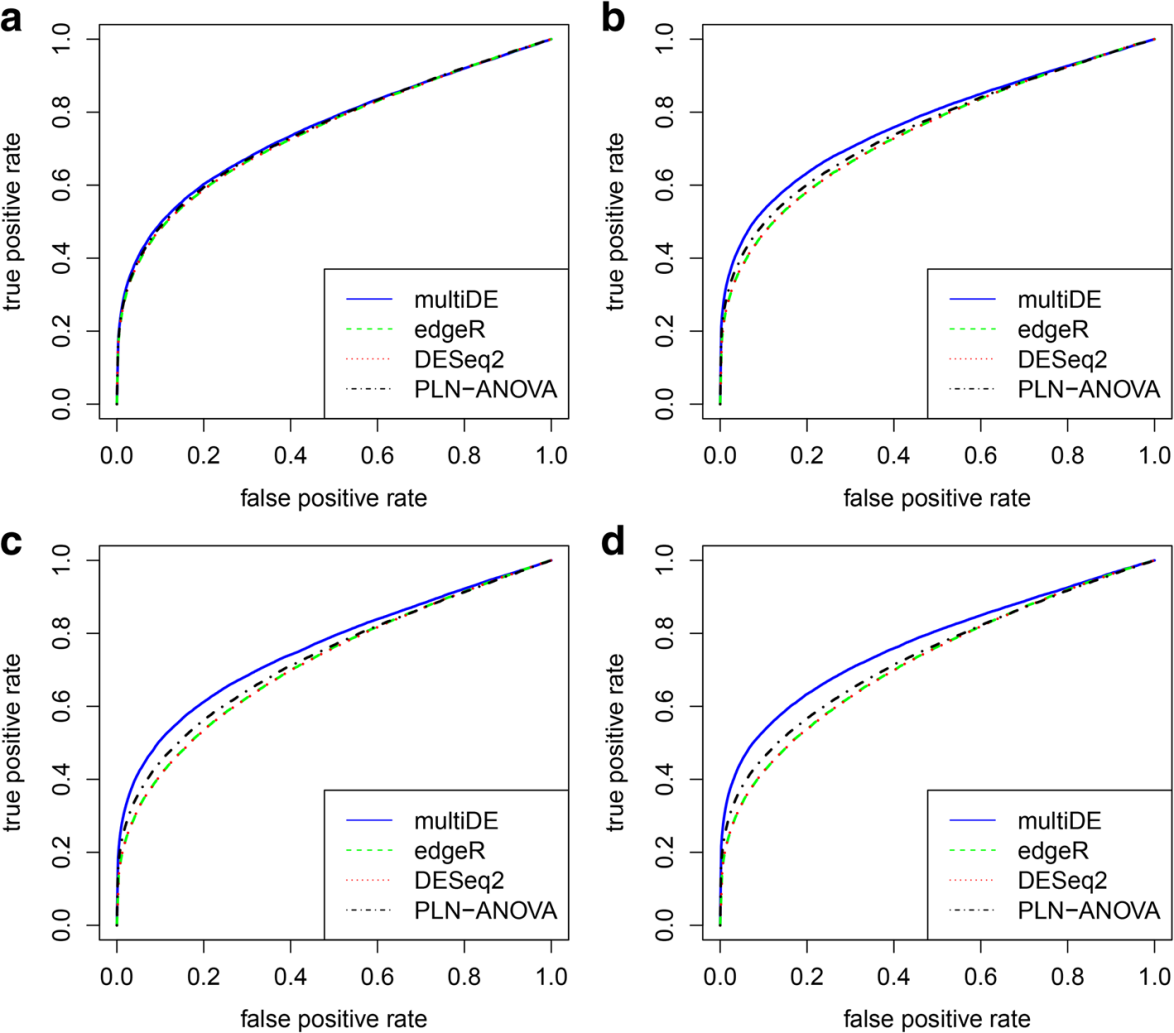
ROC curves in matched sample situation. **a** *D*=2; **b** *D*=3; **c** *D*=4; **d** *D*=5

**Fig. 8:**
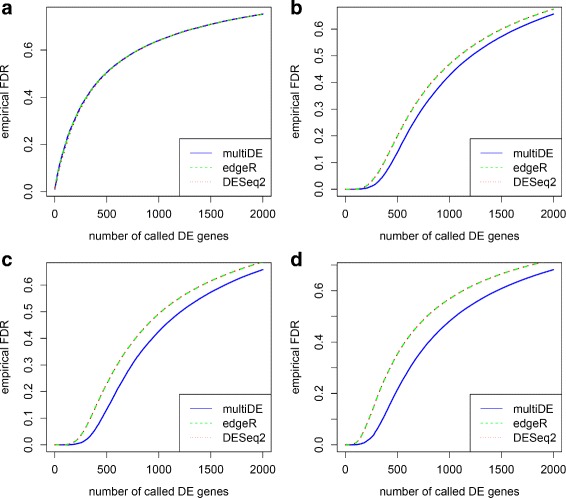
False discovery rates in unmatched sample situation. **a** *D*=2; **b** *D*=3; **c** *D*=4; **d** *D*=5

**Fig. 9:**
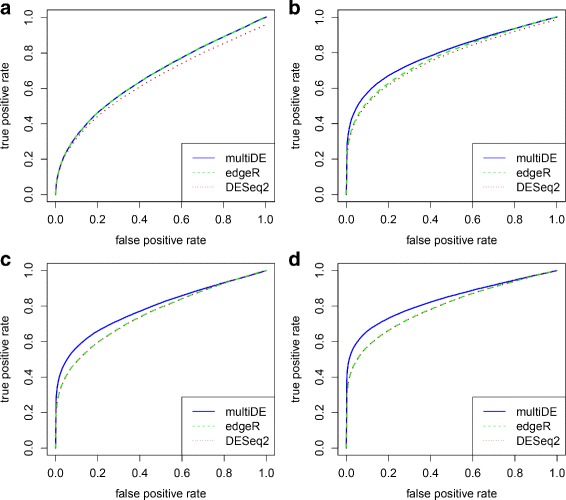
ROC curves in unmatched sample situation. **a** *D*=2; **b** *D*=3; **c** *D*=4; **d** *D*=5

We obtained the *p*-values for testing *H*_0_:*v*_*g*_=0 using the four considered methods (PLN-ANOVA was applied only in the matched sample situation), and evaluated the performance of these methods through ROC curves and FDRs (Figs. 6, 7, 8 and 9). For *N*=1,…,2000, we evaluated the FDRs of top *N* identified DE genes. The FDR-adjusted *p*-values were calculated using the R function *p.adjust*, and the empirical FDRs were consequently obtained. In terms of FDRs, the four methods performed quite comparably when *D*=2. On the other hand, multiDE and PLN-ANOVA had smaller FDRs compared with edgeR and DESeq2 when *D*>2. According to the ROC curves, when *D*=2, multiDE was comparable with the other two methods in the unmatched sample situation, and the former was even slightly more powerful in the matched sample situation. When *D*>2, multiDE was evidently more powerful than the other methods including PLN-ANOVA, and the power gain steadily went up as the number of conditions increased.

#### Sensitivity analysis

**Fig. 10:**
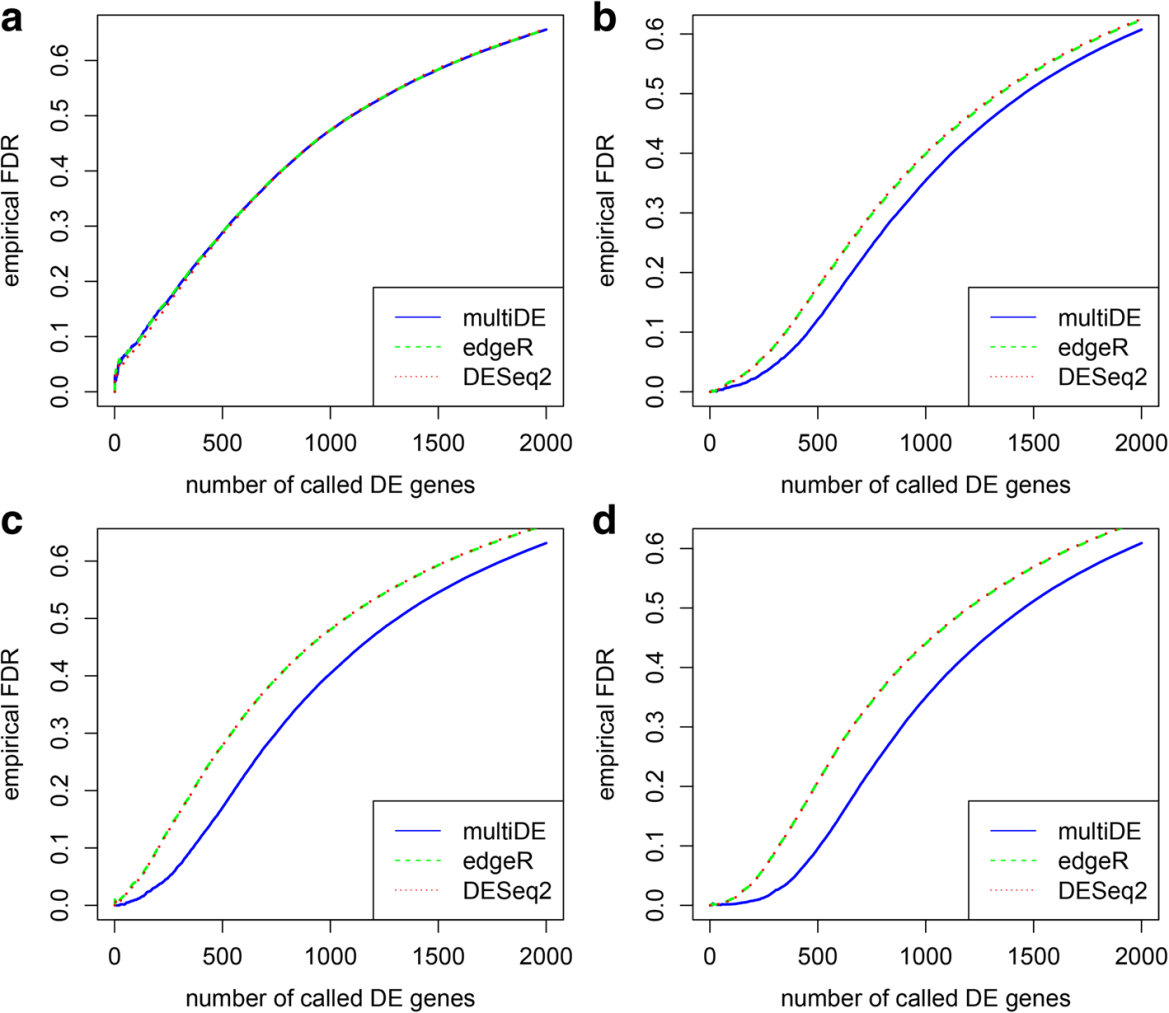
False discovery rates for model sensitivity analysis. **a** *D*=2; **b** *D*=3; **c** *D*=4; **d** *D*=5

**Fig. 11:**
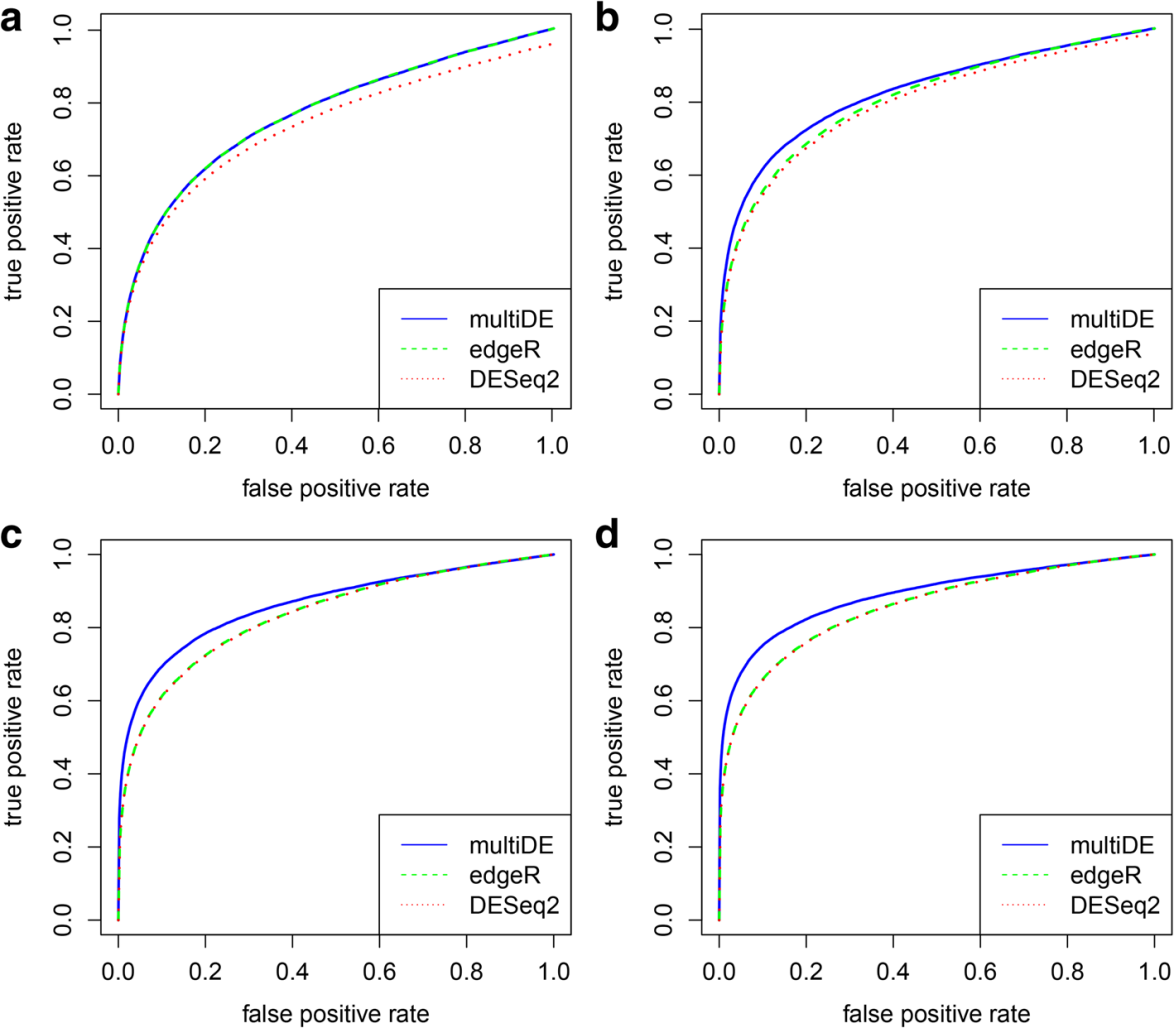
ROC curves for model sensitivity analysis. **a** *D*=2; **b** *D*=3; **c** *D*=4; **d** *D*=5

The proposed method multiDE is based on model (1). In real situations, this model might not hold. Therefore, we conducted a sensitive analysis by generating data via the following model:

21 $$ \log \mu_{dg}=\mu+\alpha_{d}+\beta_{g}+\gamma_{dg},  $$

where *γ*_*dg*_ was generated from the uniform distribution on the interval (−0.75,0.75) for genes 9,001 through 10,000. Other parameters setting and data generation process were the same as before. We only present the results for unmatched sample situation since the results for matched sample situation were similar. Again, multiDE outperformed edgeR and DESeq2 in terms of both FDRs and ROC curves (Figs. 10 and 11). Interestingly, model misspecification did not alter the performance advantage of multiDE over the other methods.

### Real data analyses

In order to evaluate their relative performance on real data, multiDE, edgeR, and DESeq2 were applied to data from two biological experiments with multiple treatment conditions. Furthermore, PLN-ANOVA was applied to the first dataset with matched samples. In each of these two experiments, only a single sample was sequenced in each lane, so we only used between-lane normalization methods.

#### Psoriatic study

In this study, the major interest was to detect the influence of aryl hydrocarbon receptor (AhR) on RNA expression profiles of psoriatic lesion cells [18]. Each of eight patients were treated with culture treatment of DMSO (vehicle control), AhR agonist FICZ, and AhR antagonist CH-2233191. RNA-seq data were obtained using Illumina Genome Analyzer II platform for each of three treated lesion tissue samples. Therefore, this was a matched sample design. The RNA-seq read counts were derived from the GEO database (accession ID: GSE47944). We kept 13,416 genes with maximal read counts greater than 50 in each treatment condition.

With multiDE, the estimated *u*_*d*_ for the three conditions (vehicle control, AhR agonist, and AhR antagonist) were 1, −4.20, and 3.20, respectively, which coincided with the fact that AhR-activating ligands reduced inflammation in the lesion of psoriasis patients and AhR antagonists upregulated inflammation.

**Fig. 12:**
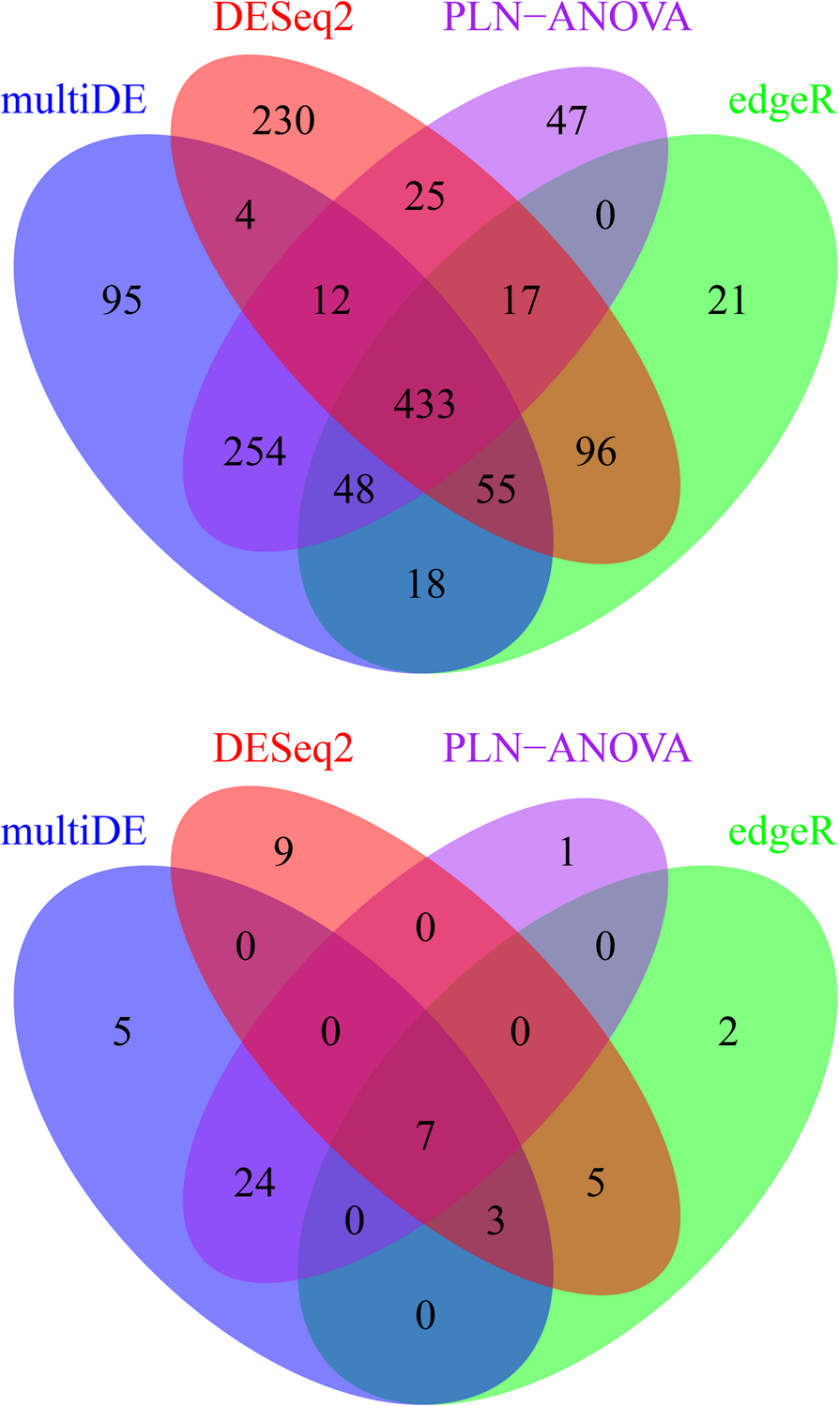
Number of identified DE genes in the psoriatic study. *Upper panel*: without Bonferroni adjustment; *Down panel*: with Bonferroni adjustment

At significance level 0.05, multiDE, PLN-ANOVA, edgeR, and DESeq2 identified 919, 836, 688, and 872 DE genes, respectively. After Bonferroni adjustment, multiDE, PLN-ANOVA, edgeR, and DESeq2 identified 39, 32, 17, and 24 DE genes, respectively (Fig. 12). Evidently, multiDE detected most DE genes. Of the five genes (BATF2, HRNR, SIGLEC1, SLC4A11, CXCL10) uniquely identified by multiDE (with Bonferroni adjustment), four were found to be closely related to psoriasis. In detail, BATF2 could induce the development of CD8 *α*+ dendritic cells, the most powerful antigen presenting cell during inflammation [19]. The up regulation of HRNR, which encoded granular layer keratin bundling proteins, was closely related to psoriatic lesions [20]. As a chemoattractant of a serial of immune cells, encoded protein of CXCL10 also played an important role in psoriatic immune abnormality [21].

It is well known that housekeeping genes maintain basic cellular functions, and they are expressed in all types of cells of an organism. Some typical housekeeping genes (eg., ACTB, GAPDH, NONO, PGK1, PPIH) have relatively constant expressions in most non-pathological situations [22], which can be used to evaluate the false positive performance of DE analysis methods. We found that the aforementioned five reference housekeeping genes were not identified to be DE genes by any of the four considered methods (Table 1).

**Table 1:** *P*-values of DE analysis for five housekeeping genes in the psoriatic study

Method	ACTB	GAPDH	NONO	PGK1	PPIH
multiDE	0.1081	0.3010	0.8429	0.1925	0.5984
edgeR	0.1814	0.2139	0.9340	0.1907	0.8923
DESeq2	0.2247	0.1852	0.8984	0.1608	0.8084
PLN-ANOVA	0.1411	0.3862	0.8282	0.2563	0.6134

#### Embryonic stem cells study

The second dataset on a study of Homo sapiens hormone embryonic stem cells was downloaded from the NCBI GEO database (accession ID: GSE36552). To find causal relationship between gene expression network and cellular phenotype, Yan et al. derived embryonic stem cells from donated human pre-implantation embryos, prepared cDNA and sequenced them by Illumina HiSeq 2000 [23].

RNA-seq samples were obtained from the embryonic stem cells of nine unrelated individuals, so this was an unmatched design and PLN-ANOVA was not applicable. The embryonic stem cells were obtained at the 2-cell stage, three at the 4-cell stage, and the other three at the 8-cell stage. We aligned the downloaded RNA-seq reads to human reference genome hg19 (UCSC release) using the bioinformatics tool *TopHat* [24], and counted the reads for each gene using the Python program *htseq-count* [25]. Altogether, 6,526 genes with the maximal counts greater than 50 in each treatment condition were kept.

With multiDE, the estimated *u*_*d*_ for the three conditions (2-cell stage, 4-cell stage, and 8-cell stage) were 1, 0.94 and, −1.94, respectively, indicating that the gene expression difference between the 2-cell and 8-cell stages could be generally large, while the difference between the 2-cell and the 4-cell stages was generally minor.

**Fig. 13:**
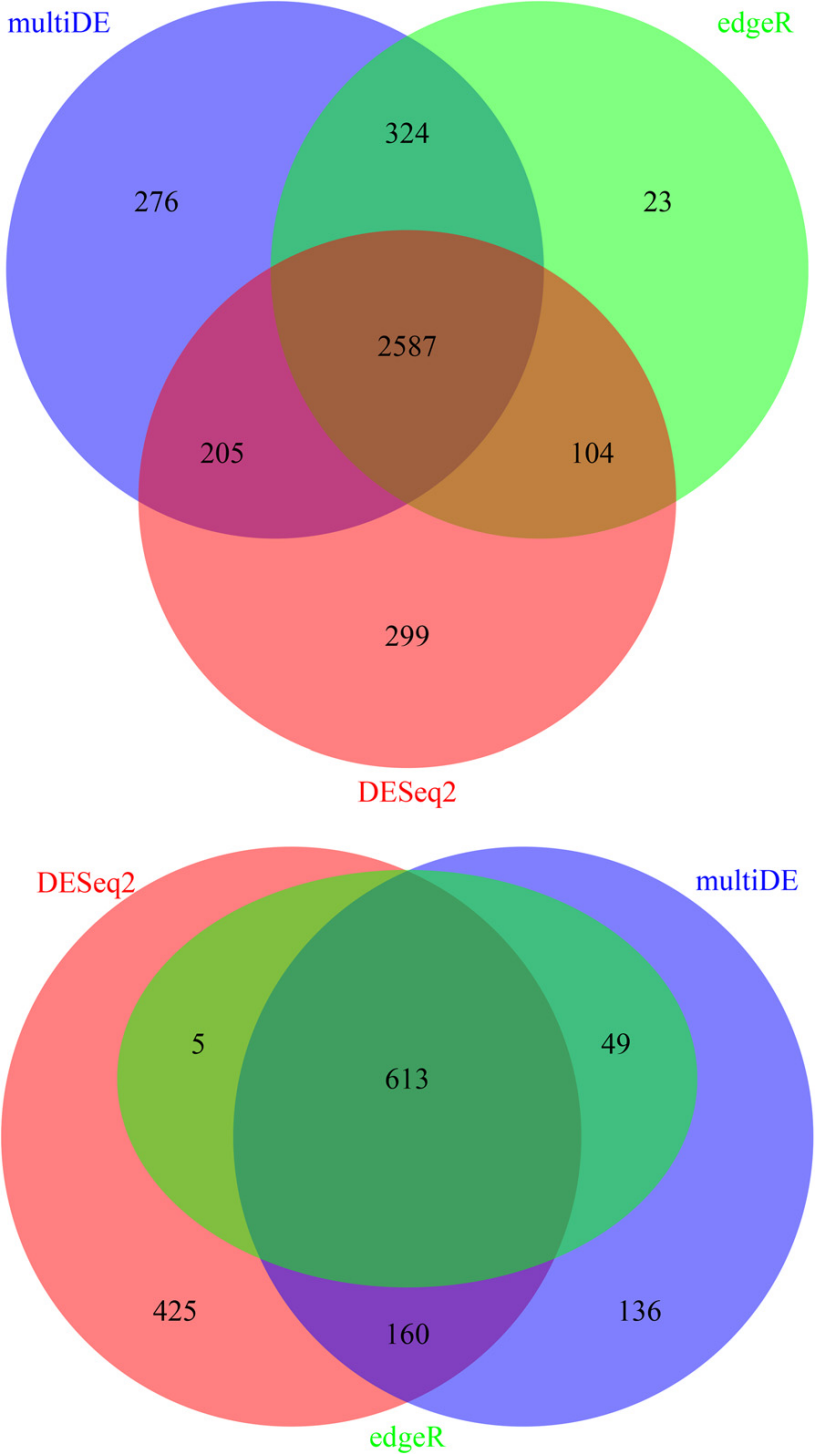
Number of identified DE genes in the embryonic stem cells study. *Upper panel*: without Bonferroni adjustment; *Down panel*: with Bonferroni adjustment

Presented in Fig. 13 are the numbers of DE genes identified by multiDE, edgeR, and DESeq2 at significance level 0.05 with or without Bonferroni adjustment. Among all three methods, multiDE detected most DE genes without Bonferroni adjustment, with a number of 3,392, compared with 3,038 and 3,195 by edgeR and DESeq2, respectively. With Bonferroni adjustment, DESeq2 identified most DE genes, with a number of 1,092, compared with 1,058 and 667 by multiDE and edgeR, respectively.

**Fig. 14:**
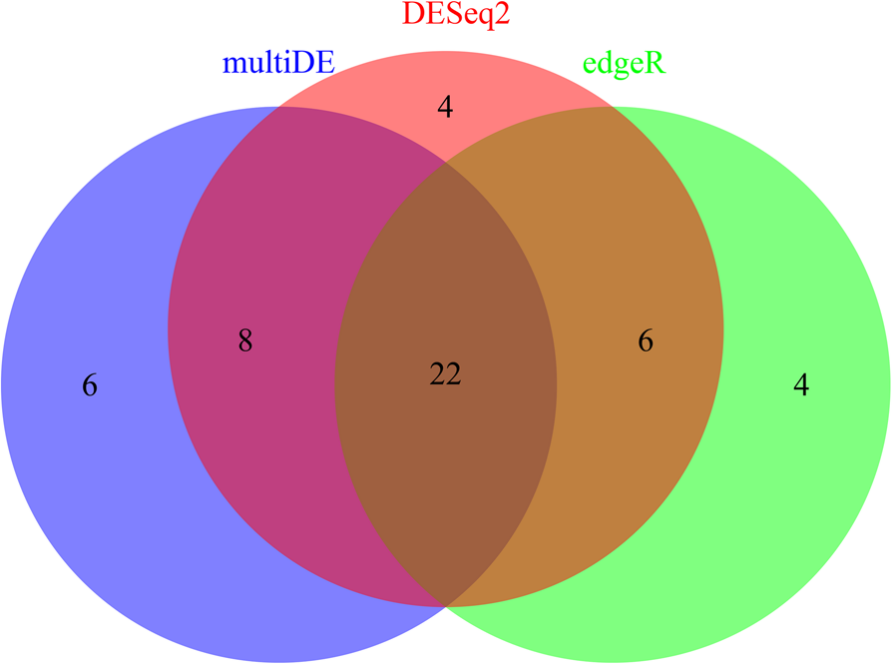
Number of detected GO terms using identified DE genes in the embryonic stem cells study

Using the identified DE genes (with Bonferroni adjustment), we then conducted GO analysis with DAVID [26, 27]. The numbers of identified GO terms are presented in Fig. 14. Evidently, multiDE enriched most GO terms, and the GO terms uniquely enriched by multiDE included protein catabolic process, protein ligase activity, and so on (Table 2). These uniquely enriched GO terms were found to be closely related to the development of embryo. First, ligases always play multiple important roles in embryo development. For instance, the deficiency of DNA Ligase IV in mice might lead to defective neurogenesis and embryonic lethality [28]. Besides, hSmurf1, a ubiquitin ligase, was shown to have the ability of controlling both embryonic development and a wide variety of cellular responses [29]. Second, the balance of metabolic and protein catabolic was in subtle poise during the development of embryo. As an alternative emergency way supplying energy, catabolism was of significant importance when embryos were facing the threat of nutrient deficiency, especially in their early stage [30]. Third, it was evident that the majority of human embryos donated for research were suffering from various cellular defects, thus the chance of innate embryo malnutrition would be greatly enhanced [31].

**Table 2:** Exclusively enriched GO terms using identified DE genes in the embryonic stem cells study

	GO code^1^	GO term description	*P*-value^2^
multiDE	GO:0030163 [BP]	Protein catabolic process	1.72e-04
	GO:0044257 [BP]	Cellular protein catabolic	5.60e-04
		process	
	GO:0005829 [CC]	Cytosol	4.00e-03
	GO:0030530 [CC]	Nuclear ribonucleoprotein	6.16e-03
		complex	
	GO:0019787 [MF]	Small conjugating protein	2.71e-02
		ligase activity	
	GO:0031967 [CC]	Organelle envelope	4.32e-02
edgeR	GO:0010468 [BP]	Regulation of gene expression	1.29e-03
	GO:0022618 [BP]	Ribonucleoprotein complex	1.27e-02
		assembly	
	GO:0044452 [CC]	Nucleolar part	1.61e-02
	GO:0006413 [BP]	Translational initiation	4.86e-02
DESeq2	GO:0000279 [BP]	M phase	1.31e-04
	GO:0005819 [CC]	spindle	3.70e-04
	GO:0030880 [CC]	RNA polymerase complex	2.07e-03
	GO:0006259 [BP]	DNA metabolic process	2.83e-03

^1^[BP], biological process ontology; [CC], cellular component ontology; [MF], molecular function ontology.

^2^Bonferroni adjusted *p*-value

As in the psoriatic study, we used the five reference housekeeping genes to verify the performance of the above three DE methods. Since the gene NONO had maximal counts less than 50 in at least one treatment condition, we excluded this gene in the DE analyses. The other four genes (ACTB, GAPDH, PGK1, PPIH) were not identified to be DE genes using multiDE and edgeR. On the other hand, DESeq2 identified GAPDH as a DE gene (Table 3), suggesting that DESeq2 had more false positive findings.

**Table 3:** *P*-values of DE analysis for four housekeeping genes in embryonic stem cells study

Methods	ACTB	GAPDH	PGK1	PPIH
multiDE	0.5419	0.1064	0.2056	0.7460
edgeR	0.5351	0.1845	0.4914	0.9021
DESeq2	0.6540	0.0151	0.6484	0.9666

## Discussion

Models for fitting the distribution of read count data are essential for detecting DE genes. In experiments involving multiple conditions, it would be of great interest to detect those genes that are differentially expressed between at least two conditions. The traditional statistical methods are generally based on an ANOVA like framework, and the number of the degrees of freedom for detecting DE genes is equal to *D*−1 (*D* is the number of conditions). In this paper, we propose to reduce the number of the degrees of freedom from *D*−1 to one based on a new dimension reduced model. The new method multiDE based on this model can handle both matched and unmatched samples. If *D*>2, multiDE greatly outpermed the existing methods in our simulation studies, even if the model used to generate data was severely misspecified.

If only a single sample is sequenced in each lane, one needs only to correct the technical bias due to library size effect since the lane-specific efect can be absorbed into the library size factors. In multiDE, there are various options for estimating size factors. Four between-lane normalization methods (i.e., MEDIAN, TOTAL, QUANTILE, and TMM) can be used to estimate size factors. In our simulation study based on a real dataset, TMM slightly outperformed the other three normalization methods. Furthermore, in [14], TMM had been shown to be robust against outlying read counts and DE genes, and it outperformed other methods in simulation studies. Therefore, we recommend TMM in real data applications. If two or more samples are sequenced in the same lane, it would be advantageous to use any within-lane normalization method before between-lane normalization [12].

In multiDE, two methods implemented in edgeR and DESeq2 can be used to estimate dispersion parameters. The two dispersion estimation methods performed comparably in our simulation studies. When estimating *u*_*d*_, the size of gene set *S* can be specified to be the number of significant DE genes (after FDR adjustment) by any existing method such as edgeR or DESeq2.

## Conclusions

In this paper, the new method multiDE is developed based on a dimension-reduced model for the purpose of detecting DE genes between multiple conditions. Through both simulation studies and real data applications, multiDE was shown to outperform the existing benchmark methods. The proposed method multiDE has been implemented in an R package. This package requires that each condition has at least two biological replications, it takes RNA-seq read counts as input data and can be used to estimate fold changes and to conduct Wald tests for detecting DE genes between various conditions. Three functions are provided in multiDE, namely *normalization*, *dispersion*, and *multiDE*, which can be used to calculate size factors using four normalization methods (i.e., MEDIAN, TOTAL, QUANTILE, and TMM), to estimate dispersion parameters using two methods provided in edgeR and DESeq2, respectively, and to calculate DE *p*-values and fold changes and their standard errors. Using a desktop computer with a 3.20GHz CPU, it took multiDE only a few seconds to analyze two real datasets.

## Abbreviations

AhR, aryl hydrocarbon receptor; DE, differentially expressed; FDR, false discovery rate; NB, negative binomial; ROC, receiver operating characteristic
